# Medoidshift clustering applied to genomic bulk tumor data

**DOI:** 10.1186/s12864-015-2302-x

**Published:** 2016-01-11

**Authors:** Theodore Roman, Lu Xie, Russell Schwartz

**Affiliations:** Computational Biology Department, School of Computer Science, Carnegie Mellon University, 5000 Forbes Ave, Pittsburgh, 15213 PA USA; Joint Carnegie Mellon/University of Pittsburgh Ph.D. Program in Computational Biology, 5000 Forbes Ave, Pittsburgh, 15213 PA USA; Department of Biological Sciences, Mellon College of Science, Carnegie Mellon University, 4400 Fifth Avenue, Pittsburgh, 15213 PA USA

**Keywords:** Computational biology, Clustering, Tumor, Heterogeneity

## Abstract

Despite the enormous medical impact of cancers and intensive study of their biology, detailed characterization of tumor growth and development remains elusive. This difficulty occurs in large part because of enormous heterogeneity in the molecular mechanisms of cancer progression, both tumor-to-tumor and cell-to-cell in single tumors. Advances in genomic technologies, especially at the single-cell level, are improving the situation, but these approaches are held back by limitations of the biotechnologies for gathering genomic data from heterogeneous cell populations and the computational methods for making sense of those data. One popular way to gain the advantages of whole-genome methods without the cost of single-cell genomics has been the use of computational deconvolution (unmixing) methods to reconstruct clonal heterogeneity from bulk genomic data. These methods, too, are limited by the difficulty of inferring genomic profiles of rare or subtly varying clonal subpopulations from bulk data, a problem that can be computationally reduced to that of reconstructing the geometry of point clouds of tumor samples in a genome space. Here, we present a new method to improve that reconstruction by better identifying subspaces corresponding to tumors produced from mixtures of distinct combinations of clonal subpopulations. We develop a nonparametric clustering method based on medoidshift clustering for identifying subgroups of tumors expected to correspond to distinct trajectories of evolutionary progression. We show on synthetic and real tumor copy-number data that this new method substantially improves our ability to resolve discrete tumor subgroups, a key step in the process of accurately deconvolving tumor genomic data and inferring clonal heterogeneity from bulk data.

## Background and introduction

One of the major challenges of understanding tumor genomics is the existence of massive heterogeneity both between patients (inter-tumor heterogeneity) and within single tumors (intra-tumor heterogeneity). Clinically similar tumors may exhibit strikingly different genomic profiles [1–3] and patterns of progression [1, 2]. Even within a single tumor, there can be dramatic differences in genomic profiles at regional [4–6] and even cell-to-cell levels [7–15]. The genomes of tumors thus exhibit significant variation from individual to individual (inter-tumor heterogeneity) as well as cell-to-cell or region-to-region within single tumors (intra-tumor heterogeneity). This heterogeneity presents significant challenges to characterizing progression of individual tumors and finding the commonalities among progression processes across tumors.

New technologies have made it possible to study tumor genomics in much finer detail, but often at the expense of creating even harder computational problems in making sense of the data. Single-cell sequencing [9, 11, 13–17] has offered the promise of reconstructing tumor genomes at the cellular level, but for the moment remains noise-prone and too costly to apply to large numbers of cells in large numbers of tumors. As a result, attempts to reconstruct tumor heterogeneity in large patient populations have so far largely relied on computational inferences using a class of method called “mixed membership modeling” (also known as unmixing or deconvolution) applied to genomic data from bulk tumor cell populations [18, 19]. Such methods interpret genomic data as mixtures of small numbers of major cell populations and seek to reconstruct the underlying cell populations from the mixed data. They were originally used largely as a way of “purifying” tumor data by separating contributions from tumor versus normal cells [20] but are now widely used to reconstruct clonal subpopulations and/or the progression among them from various genomic data sources (see, for example, [21–30]). Although such methods are now finding widespread use, they still fall far short of being able to reconstruct the true complexity of tumor heterogeneity within or between tumors that can be observed from true single-cell data [9, 13–15, 31, 32]. For a more complete review of computational challenges to reconstructing tumor heterogeneity, see [12].

Our work has pursued a particular agenda of reconstructing models of intra-tumor heterogeneity of individual tumors by taking advantage of inter-tumor heterogeneity across a population of tumors [19, 21, 33]. Specifically, we seek to construct a model of evolution of cell populations making up all of the tumors in a patient population, so as to explain each individual tumor as a distinct mixture of those inferred cell populations. In recent work [33], we showed that it was possible to produce a more nuanced analysis of the mixture composition of tumor cell populations by using the assumption that tumors falling into different subtypes or progression pathways will be composed of distinct mixtures of cell types. A key step in reconstructing these mixtures, and the focus of the present paper, is to partition a population of tumors into clusters, where each cluster of tumors is approximately explainable as a mixtures of a distinct subset of a common set of cell types. By finding these subsets of tumors prior to mixture reconstruction, we can infer more sophisticated mixture models accounting for this kind of clonal substructure and improve accuracy of mixture reconstruction. Given a clustering of tumors into subtypes, we can then apply methods for mixed membership modeling to reconstruct models of the cells defining these clusters. We can further organize these cell models into a tumor phylogeny, describing how evolution from a common ancestral cell type could lead to a spectrum of cells sufficient to explain all of the observed tumors across all of the clusters as distinct linear mixtures of those basic cell types.

Identifying these subgroups of tumors can be interpreted geometrically as a problem of partitioning point clouds of tumor genomic data into distinct low-dimensional subregions of a larger ambient genomic space of all tumors. That is, we can conceptualize tumors as points in a geometric space, where each gene or genomic location profiled yields a coordinate for each tumor. Distinct genomic profiles will then lie at distinct points in that high-dimensional space. Mixtures of different kinds of cells would be expected to form low-dimensional objects whose vertices correspond to the positions of the genomic profiles of the component cells. Distinct mixtures, then, would sit in distinct subregions of that high dimensional space, corresponding to mixtures of distinct sets of high-dimensional component vectors. The problem of finding tumors composed of distinct cell types can then be mathematically represented as the problem of finding point clouds that sit in these separate low-dimensional subregions. That problem itself can be posed as a special form of clustering problem. Many clustering methods have been used to study tumor genomic data, including k-means and similar methods, hierarchical methods, and Gaussian mixture models (see, for example, [34]). All of these methods are designed, however, for the problem of partitioning a high-dimensional data set into disjoint point clouds, not into discrete lower-dimensional subregions within a single contiguous point cloud.

To pose the problem more formally, we can think of each tumor sample *x* as a vector of genomic profiles (expression or copy number levels) at *n* genomic sites, (*x*_1_,…,*x*_*n*_). Our model depends on a hypothesis that distinct tumors will approximately evolve along similar progression pathways and thus evolve similar cell types over the course of their progression. We can then conceptualize each tumor *x* as a noisy mixture of a subtype of a genomic profiles of a common set of cell types *c*_1_=(*c*_11_,…,*c*_1*n*_),…,*c*_*k*_=(*c*_*k*1_,…,*c*_*kn*_). Any given tumor can then be modeled as a linear combination of those cell types *x*≈*a*_1_*c*_1_+*a*_2_*c*_2_+…+*a*_*k*_*c*_*k*_. This would in principle cause tumors to be approximately confined to a *k*-dimensional space, as is assumed in our prior unmixing approaches [19, 21]. Because tumors cluster into distinct subtypes defined by similar progression pathways, though, we would expect different tumors to sample from different subsets of that common set of cell types and thus to have different subsets of non-zero *a*_*i*_ coefficients. If a given tumor subtype evolves a subset of *k*^′^ of those *k* cell types in its progression, then tumors of that subtype will approximately occupy a *k*^′^-dimensional subspace of the *k*-dimensional space defined by linear combinations of the full set of cell types. Our goal in the present work is to partition tumors into clusters that appear to occupy these low-dimensional subregions called subspaces so that we can more accurately infer the genomic profiles of the *k*^′^ cell types corresponding to each cluster and thus build an accurate mixture model of each cluster.

In the present work, we tackle this problem of better decomposing genomic point clouds into low-dimensional subspaces for the purpose of improving deconvolution of cell subpopulations within mixed data sets. Specifically, we seek to partition a point cloud of tumors in a high-dimensional genomic space into subsets of tumors that occupy distinct low-dimensional subspaces and that can be interpreted as being composed of distinct cellular subpopulations. We use an approach based on medoidshift clustering [35], a non-parametric method for unsupervised clustering that is well suited to working with the sparse, high-dimensional point clouds characteristic of tumor genomic data. We adapt the medoidshift method to the present problem using a novel distance measure and two-stage kernel strategy designed to deal with the high noise of genomic data and the non-standard application to discovering discrete subspaces of a contiguous geometric object.

## Methods

### Overview of medoidshift

Medoidshift clustering is a method within the class of mode–seeking clustering algorithms [35]. It may also be considered a modification of meanshift clustering [36] that allows for cluster centers that are data points (which may be integral to data interpretation), as well as faster runtime in the case of the addition of data to a set for which medoidshift has already been computed [35]. Medoidshift clustering works by finding local centers of density as follows. Assume the data $X \in {\mathbb {R}}^{m \times n}$ consist of *m* data points in an *n*-dimensional ambient space. Then for a shadow kernel function *Φ*(·), and neighborhood size $h \in {\mathbb {R}}$, and scaling constant $c_{o} \in {\mathbb {R}}$, we can define a measure of the contribution of a point to the center of mass of another point. For a data point *x*∈*X*, we can define the kernel density estimator *f*(·) at that point by

(1) $$ f(x) = c_{o} \sum_{i=1}^{m}{\Phi\left(\left|\left|\frac{x-x_{i}}{h}\right|\right|^{2}\right)}  $$

as described in [35], where *c*_*o*_ is a scaling constant, *Φ*(·) is as described in [35], and $\left |\left | {\cdot } \right |\right |$ is a distance function. Medoidshift clustering works by assigning a representative to each putative cluster using the data point nearest the center of mass of a neighborhood of points (i.e., the centroid of the neighborhood). We can therefore set the representative of *x*, say *y*, as follows, using the same notation as our previous equation:

(2) $$ y_{k+1} = \underset{y \in N(x)}{\arg\min}{\sum_{i=1}^{m}{||x_{i} - y|| \phi\left(\left|\left|\frac{x_{i}-y_{k}}{h}\right|\right|^{2}\right)}}  $$

where *N*(*x*) is the neighborhood of *x*, *y*_*k*+1_ is the (*k*+1)^*st*^ candidate for medoid, *y*_*k*_ is the *k*^*th*^ medoid candidate, *ϕ*=−*Φ*^′^, and other variables are defined as above. *y*_0_ can be any point within *N*, although in practice we choose the point with smallest distance to *x* in the kernel space. Sheikh et al. [35] offers a proof of convergence for the algorithm, as well as a more detailed outline of medoidshift in general. We note that strictly speaking some kernel function is always applied to the data; however, in the case that we use a linear kernel where Euclidean distances hold, for the sake of simplicity in reference, we call this approach “no kernel” or “kernel-free”.

For the present applications, on both synthetic and real tumor data, we have chosen a hyperparameter neighborhood size of 1 (i.e., *h*=1), and scaling factor of 1 (i.e., *c*_0_=1), given that we can normalize data to the [ 0,1] range, because *h*=1 encodes the belief that each cluster is approximately unit length in the space in which it is embedded. In other words, by setting *h*=1, we allow points within unit distance, which may or may not be transformed using a kernel function, to strongly influence the choice of medoid for a point, but decrease the influence for points outside that neighborhood. For unit length clusters, this minimizes the influence on centroids for points outside that cluster. Additional preprocessing and normalization required for the real data is described in Subsection 2.4. “Validation on real tumor data”.

### Two-stage medoidshift clustering

Converting medoidshift into a method for decomposing a point cloud into distinct subspaces, rather than spatially separated clusters, can be accomplished with an appropriate choice of distance measure and kernel function to ensure low distance between tumors in a common subspace and high distance between those in distinct subspaces, even if they are close in the ambient genomic space by more conventional Euclidean distances. A similar problem is encountered by more general manifold learning methods. One popular method for reconstructing low–dimensional geometries embedded in higher–dimensional ambient spaces and measuring the distances in the planes and hyperplanes of the embedded manifolds is ISOMAP [37], which uses path distances in a nearest-neighbor graph to measure distance between points on low-dimensional manifolds defined by data in a high-dimensional ambient space. Tumor data would be expected to largely conform to the assumptions of the ISOMAP method [37, 38]. However, ISOMAP has been shown to have difficulty when the data occupy a low-dimensional manifold but are perturbed by ambient-dimensional noise [39], a property that is characteristic of the tumor genomic data we seek to resolve [40, 41]. We apply an approach based on a similar strategy to ISOMAP [37] for identifying low-dimensional manifolds based on path distances in a neighbor graph of the data points, but intended to deal better with the noise characteristics of genomic data.

Our approach uses a two-stage strategy to balance the desire for ISOMAP-like manifold reconstruction with tolerance to genomic noise characteristics. In the first stage we use kernel-free clustering with squared Euclidean distance intended largely to suppress noise in the data. In this stage, representatives of the data points are a reduced set of data points representing local centers of mass in the ambient space. Because medoidshift clustering is a mode–seeking algorithm [35], the representatives of points determined by medoidshift clustering after one round will be nearer to the more dense regions of the data cloud. Under our model assumptions — unbiased noise and noise variance small relative to the signal — the representatives after the first round should constitute a denoised version of the original data. Prior work [42] shows that these assumptions are reasonable for genomic copy number data, which appears to exhibit unbiased noise with variance on the order of 10 percent of the signal.

In the second stage of clustering, we use a kernel function *K*, where, *K*=1−*e*^*D*/*h*^, where *h* is a scalar bandwidth and *D* is the distance between points. Distances are measured as shortest path length in the point neighbor graph, in a similar fashion to ISOMAP [37], with distances between neighboring points measured by the square of the Euclidean distance. We begin with the representatives derived from the end of the first, kernel-free stage of medoidshift clustering, and use the kernel-based medoidshift clustering until convergence to yield a small set of final representatives. The negative exponential kernel function has the effect of causing the clustering to identify extreme points of the point cloud in different subspaces as cluster representatives, rather than centroids in the more conventional sense. The combination of the specialized kernel and the square Euclidean path distance transforms the clustering into an approach for identifying points in different subspaces rather than those isolated in conventional Euclidean space. By itself, though, this specialized clustering is very sensitive to outliers in the data. Under the interpretation that the result of the first stage yields a denoised version of the data, however, the second stage will decompose the underlying geometry of the denoised point cloud in order to assign individual tumors to discrete subspaces, interpreted to represent distinct evolutionary trajectories among the possible progression pathways of the tumors.

All applications of medoidshift clustering in the present work were run on a desktop workstation with an Intel Core i7-4770K processor at 3.5 GHz per core, 32 GB of RAM, and Matlab running in 64–bit mode on Ubuntu.

### Validation on synthetic data

We generated seven scenarios of synthetic data to correspond to different possible tumor progression models, each involving small numbers of possible states of progression along a tumor phylogeny capturing the progression pathways of a patient population. For each synthetic data set, we begin by defining a tumor phylogeny: an evolutionary tree describing how a set of cell types might evolve by progression from a common ancestral cell type. Each such phylogeny defines a mixture scenario, in which each path from root to leaf in the phylogeny corresponds to a mixture of cell types we would expect to see in a subtype of tumors. The length of the path will define the dimension of the corresponding point cloud (one dimension per cell type in the mixture). Sharing of cells between paths will manifest as sharing of basis vertices between subspaces. The scenarios are illustrated in Fig. 1, which provides, for each scenario, a line-graph icon describing its simplicial complex structure and a representation of the progression pathways to which that simplicial complex structure corresponds. For example, Fig. 1a, derives from an evolutionary scenario in which an initial progenitor cell type evolves to an early progression state which then evolves two late progression child states. That scenario results in two tumor subtypes sharing the progenitor and early progression state but differing in their late progression state. Geometrically, that mixture scenario would yield two three-dimensional (triangular) simplices sharing a two-dimensional edge. The seven scenarios are as follows:

1. Two triangles sharing an edge (Fig. 1a)

**Fig. 1:**
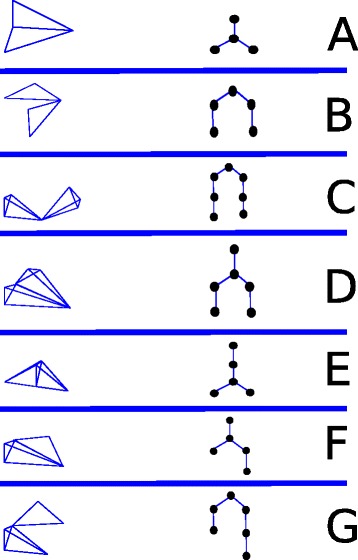
Visual representation of geometric structures tested with synthetic data and corresponding evolutionary scenarios
2. Two triangles sharing a point (Fig. 1b)
3. Two tetrahedra sharing a point (Fig. 1c)
4. Two tetrahedra sharing an edge (Fig. 1d)
5. Two tetrahedra sharing a face (Fig. 1e)
6. A triangle and a tetrahedron joined at an edge (Fig. 1f)
7. A triangle and a tetrahedron joined at a point (Fig. 1g)

For each scenario, we generated 100 replicates with each replicate consisting of 500 points in 10 dimensions, chosen to approximate the real data after application of PCA. We varied the noise standard deviation from 0 (no noise) to 0.2 in increments of 0.05. This increasing noise was to allow us to examine the performance of the approaches in the neighborhood of the estimated 0.1 standard deviation noise of real data [42]. We then analyzed the data with three variants of clustering: medoidshift with no kernel function, medoidshift using the kernel function for every step of the medoidshift, and our proposed two-stage medoidshift. We used the ISOMAP-derived distance measure for both the no-kernel function and kernel function medoidshift clusterings. A more detailed description of the simulation technique is provided below.

#### Simulation procedure

To simulate a scenario in a *p*-dimensional principal components space, we first define a set of *k* basis vectors $B \subset \mathbb {R}^{p}$, representing genomic profiles of the cell types for that scenario. We can then describe a subsimplex in terms of a subset $b = (b_{1}, \ldots, b_{k'}), \subset B$ of the basis vectors, representing the subset of tumors using a particular subset of *k*^′^ of the cell types. We then generate a sample tumor from such a cell population by generating a set of mixture fractions representing the amount of each cell type in each tumor. For a given tumor *i* we generate a mixture fraction *f*_*ij*_ for each of the *k*^′^ cell types in *b* by defining *f*_*ij*_∼*unif* [ 0,1]. We can then generate noiseless samples for each tumor *i* and gene *j* as follows:

(3) $$ y_{ij} = \frac{\sum_{r=1}^{k'} f_{ir} b_{rj}}{\sum_{r=1}^{k'} f_{ir}}  $$

**Fig. 2:**
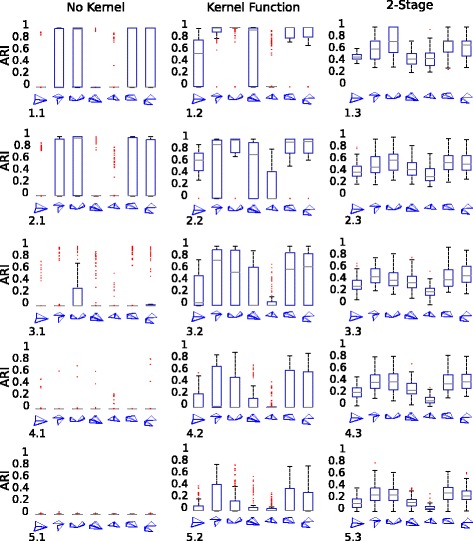
Adjusted Rand indices (ARIs) for 100 replicates of synthetic data under seven mixture scenarios with varying noise. The first column (*panels x.1*) shows the performance of medoidshift without a kernel function; the second column (*panels x.2*) show the performance of using the negative exponential kernel function; and the third column (*panels x.3*) is our new 2-stage medoidshift clustering method. Each row has increasing noise; the first row (*panels 1.y*) has no noise, the second row has *σ*=0.05 noise added, the third row has *σ*=0.1 noise added, the fourth row has *σ*=0.15 noise added, and the fifth row has *σ*=0.2 noise added

In order to simulate error in the measurements, we add then noise to the samples. For a noiseless sample *y*_*ik*_, we construct a noisy sample *x*_*ij*_ by adding 0-biased normal noise in each dimension. That is, for $j = 1,\dots,10, x_{\textit {ij}} = y_{\textit {ij}} + \mathcal {N}(0,\sigma)$, where we enumerate over *σ*∈{0,0.05,0.1,0.15,0.2} to represent the increasing noise scenarios visualized in Fig. 2.

We assessed accuracy of cluster assignment using the adjusted Rand index [43–45], a measure of cluster quality chosen because it deals well with situations in which the number of clusters is not known a priori and may differ between the ground truth and across inferences being assessed. The adjusted Rand index is computed as follows. Assume we have a known ground-truth partition of the data consisting of *n* data points; call this partition *X*=*X*_1_,*X*_2_,…,*X*_*r*_, where there are *r* total clusters in the ground truth. Suppose we have a partition of the data generated by the algorithm we are testing; call this partition *Y*=*Y*_1_,*Y*_2_,…,*Y*_*s*_, where there are *s* total clusters in the partition generated by the algorithm we are assessing. Then we can construct a matrix *N* to describe the amount of overlap in each of the clusters in *X* by rows and each of the clusters in *Y* by columns. More specifically, for entry *n*_*ij*_∈*N*, where *n*_*ij*_ is the *i*th row, *j*th column, $n_{\textit {ij}} = |X_{i} \cap Y_{j}|$. Finally, to account for the sizes of the clusters in the ground truth and found from the algorithm we would like to assess, we can use a variable to describe the sizes of the clusters. Let *a*_*i*_,∀*i*∈1…*r* be $\sum _{j = 1 \ldots s}(n_{\textit {ij}})$. Similarly, let *b*_*j*_,∀*j*∈1…*s* be $\sum _{i = 1 \ldots r} (n_{\textit {ij}})$. Then we can represent the adjusted Rand index of the algorithm we are testing using the following formula:

(4) $${} ARI = \frac{\sum_{i,j}{{n_{ij}}\choose{2}} - \left(\sum_{i} {{a_{i}}\choose{2}} \sum_{j}\left({b_{j}}\atop{2}\right)\right)/{{n}\choose{2}}}{\frac{1}{2}\left(\sum_{i}{{a_{i}}\choose{2}} \sum_{j}\left({b_{j}}\atop{2}\right)\right)-\left(\sum_{i}\left({a_{i}}\atop{2}\right)\sum_{j}\left({b_{j}}\atop{2}\right)\right)/{{n}\choose{2}}}  $$

### Validation on real tumor data

We examined two real tumor data sets, both taken from the Cancer Genome Atlas (TCGA) data repository [3], one of high-grade serous ovarian adenocarcinoma (OV) data [46] and one of lung squamous small cell carcinoma (LUSC) [47]. We applied our method to array comparative genomic hybridization (aCGH) data sets profiling copy number variation data for each tumor. We favored copy number data over expression data because copy numbers, as a measure of DNA rather than RNA, are less prone to confounding effects of cell-cell communication and from contamination by normal stromal cells [21]. aCGH data also yield relatively low noise levels, approximately 10 percent of the signal [42], which fits into the range of noise levels at which our mixture reconstruction method was previously found to provide a substantial improvement over other approaches [33]. In each case, we restricted our analysis to the autosomal chromosomes.

After downloading the copy number (CN) data, we pre-processed and compressed the data in order to load the entire dataset into memory on a workstation. Each data file from TCGA contains calls for copy number alterations, in the form of the *log*2 ratio of the alteration in the copy number of the DNA compared to normal, as well as the start and end locations for the call, and the chromosome on which the call is made. For each dataset, we scanned through each base pair, and whenever an amplification in copy number was observed in a sample, we designated a new unit, which we called a block. Within a block, then, each sample has a constant copy number. Then, for each chromosome in a dataset, we could represent the data in the form of an *m*×*n* matrix *M*, where there are *m* samples, and *n* blocks for that chromosome for that dataset. We then used principal components analysis (PCA) [48] to reduce the dimension of the data to the top 10 principal components (PCs), a way of increasing the point density, as is required by mode-seeking algorithms such as medoidshift to determine representatives [35]. Ten dimensions were chosen based on past experience that this is approximately the upper limit for the number of distinct mixture components this class of method will be able to resolve from data sets of a few hundred tumors. In order to further normalize the data into the range [ 0,1] for each dimension, which is built into the model as an assumption with respect to the neighborhood examined for each data point, we peformed the following computation:

(5) $$ \hat{M}_{i,j} = \frac{M_{i,j}-\underset{s}{\min{M_{s,j}}} }{\underset{q}{\max{M_{q,j}}} -\underset{r}{\min{M_{r,j}}} }  $$

where $\hat {M}_{i,j}$ is the normalized value, and *M*_*i*,*j*_ is the *ith* row and *jth* column of the matrix *M* described above. To encode all of the data of the genome into one unit, we then concatenated each chromosome’s block information together, where each block in each chromosome represented additional features (columns). In terms of the data matrices *M* described above, if we let *M*^*i*^ represent the matrix *M* above for the *ith* chromosome, we construct a data matrix *D*, where *D* is the horizontal concatenation of *M*^*i*^, ∀*i*=1…22, for each of the 22 non-sex chromosomes.

The ovarian cancer data set, obtained on 15 July 2015 from TCGA [3], consisted of 472 samples with both clinical and CN data. Blocking the CN data and taking the top 10 PCs of the data yielded a 472 by 10 matrix. The lung squamous small cell carcinoma dataset used was obtained from TCGA [3] on 21 July 2015, consisting of 408 samples with both clinical and CN data. Blocking the CN data and taking the top 10 PCs of the data yielded a 408 by 10 matrix.

To assess how the different clusters found in the data might become manifest in clinical data, we constructed Kaplan–Meier curves and applied the log-rank statistic to test significance of the correlation between cluster assignment and censored survival [49] for each real data set.

For the OV data, we further examined correspondence of our cluster assignments to those of the TCGA, which identified four tumor OV subtypes based on analysis of RNA expression data: differentiated, immunoreactive, mesenchymal, and proliferative. We tested significance of the overlap between our clustering and their subtyping by chi-squared test.

We further conducted a functional genomic analysis to better understand the possible biological significance of the clusters. We first converted the block coordinates back into the corresponding chromosome coordinates and then used BioMart [50] to find lists of elevated genes for each cluster. For each cluster, we used the cluster representative as determined by the two-stage clustering. For each representative, we identified each block showing at least 4-fold amplification as a block of interest at a 10kb resolution. We used Biomart [50] to extract a list of genes overlapping each block and pooled genes across blocks for each cluster. We applied DAVID [51] with default parameters to identify ontology terms overrepresented in the resulting gene lists, identifying the top cluster of DAVID functional annotations for each of the clusters found through our method.

## Results

### Synthetic data

We first applied our methods to synthetic data generated from the seven scenarios of Fig. 1. The adjusted Rand indices (ARIs) for each trial appear in Fig. 2. Each row of the figure shows results for all three cluster variants — no kernel, kernel, and our proposed two-stage method — across all seven scenarios at a single noise level, with noise increasing on each successive row. The kernel-free medoidshift was essentially unusuable on the synthetic data, with ARI at or near zero (no correspondence of true to inferred clusters) for all scenarios at modest noise levels. Even in the noise-free case, the kernel-free method is only occasionally able to produce non-zero ARI. The one-stage kernel-based method is reasonably effective in most scenarios at the lowest noise levels, but with very high variance even for modest noise. At higher noise levels, it, too, usually yields mean ARI at or near zero. Our proposed two-stage method yields far more consistent results. While it somewhat underperforms the one-stage kernel method in noise-free case, it is far less sensitive to increases in noise. The two-stage method gives qualitatively similar results across noise levels, with modest decreases in accuracy at each increasing noise level. Furthermore, the two-stage method yields far lower variances across replicates, suggesting it is meeting the goal of suppressing the high sensitivity to outliers produced by the negative-degree kernel function alone.

We also evaluated run time on the synthetic data. Mean run times across all scenarios and noise levels for each method are 1.2854 s, 1.2914 s, and 0.0121 s for no kernel medoidshift, kernel medoidshift, and 2-stage medoidshift respectively. The 2-stage method requires less time because the costly path-distance computations needed to derive the ISOMAP distances are only computed for cluster representatives after the first step of the two-step method, when the number of data points has been significantly reduced. The no-kernel and kernel techniques, by contrast, must compute the path costs from all points to all points.

### Real tumor data

Experimental data analyzed in the manuscript were taken from The Cancer Genome Atlas [3]. Contributing centers for LUSC and OV were Harvard Medical School (LUSC and OV). Copy-number data were collected using the Aglient Human Genome CGH Microarray 244A (OV) and Aglient Human Genome CGH Custom Microarray 2x415K (LUSC). The experimental procedures used in collecting these data are as described in the primary literature from the TCGA Research Network [3].

**Fig. 3:**
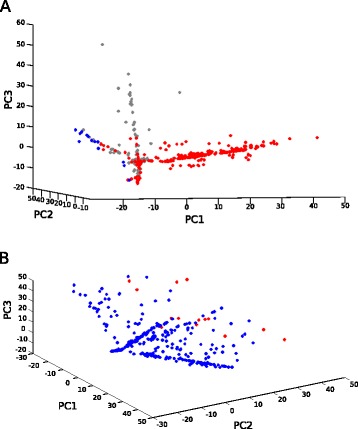
Visual representation of ovarian tumor data (OV) in principal components space (panel (**a**)), and of lung squamous small cell carcinoma (LUSC) (panel (**b**)). Data are colored based on their cluster membership as determined by 2-stage medoidshift clustering

We next analyzed the two real tumor data sets, beginning with the ovarian cancer (OV) TCGA data. We performed two–stage medoidshift clustering on the matrix of PCs of CN data, which yielded three clusters. Figure 3a shows the point cloud and cluster assignment, visualized in the space of the first three PCs. The OV data show a clear simplicial substructure, approximately described as three arms projecting from a common center. We would expect that the center would correspond approximately to normal diploid cells and the arms to mixtures of normal cells with three progression subtypes. The clustering corresponds generally, although not perfectly, to the arm structure, as intended.

To assess whether the clustering is biologically significant, we performed Kaplan-Meier survival analysis [49] to test for significant differences in survival time between pairs of clusters. We found that a comparison of cluster 1 to cluster 2 yielded a log rank test statistic chi–squared value of 12.5571, cluster 2 to cluster 3 yielded a chi-squared value of 50.5965, and cluster 1 to cluster 3 yielded a chi-squared value of 180.9646. All of the chi-squared values are strongly significant (*p*<0.01), suggesting that the clustering into subsimplices of the simplicial complex does capture a clinically significant subgrouping of tumors.

We further compared our clusters to assignments of four molecular subtypes (differentiated, immunoreactive, mesenchymal, proliferative) derived as part of the TCGA data analysis [46] by non-negative matrix factorization clustering (NMF) [52] applied to RNAseq expression data. We obtained the TCGA subtyping information from Verhaak et al. [53]. We established a contigency table of tumors comparing our cluster assignments to the TCGA subtypes, excluding 14 samples for which no subtyping label was provided and found a *χ*^2^ value of 15.48091566, yielding a weakly significant *p*-value of 0.0168. Our cluster 1 associated most strongly with the mesenchymal subtype and cluster 3 with the proliferative subtype, with cluster 2 too small for any association to be apparent.

Further ontology analysis via DAVID [51] on each of the clusters yielded enrichment for a variety of terms. In the interest of space, we focus on the most significant term grouping identified DAVID for each of our clusters. Table 1 shows these DAVID term for each of our clusters, with highly similar terms merged. We then sought to tie the enriched terms to existing analysis in the literature. The terms found appear consistent with activation of genes associated with epithelial differentiation in cluster 1, a set of pathways previously observed to be active in subsets ovarian tumors [54]. Cluster 2 shows genes that may be consistent with an immune-active subtype, although the cluster is too small for results to be significant. The top cluster 3 terms seem most associated with cytoskeletal structure and movement, which would be consistent with the proliferative subtype identification but could have many other interpretations. We also searched for correspondence between pathways dysregulated by the genes of interest from the TCGA findings [46] and pathways associated with genes found in our cluster amplicons as assessed by Entrez description [55]. Anecdotally, we found genes in several key pathways identified by the TCGA study, including NOTCH signaling and Wnt/ *β*-catenin signaling [46], although we cannot attach significances to these assignments.

**Table 1:** Summary of DAVID ontology terms most strongly associated with each ovarian (OV) and lung (LUSC) cluster

Cancer type	Cluster number	Terms
OV	1	Keratinization, small proline-rich, epidermal cell differentiation,
		epithelial cell differentiation
OV	2	Antigen processing, MHC class II, asthma, allograft rejection,
		type I diabetes mellitus, cell adhesion
OV	3	Keratin, coil 1a/b/2/12, intermediate filament, cytoskeleton,
		non-membrane-bound organelle
LUSC	1	Zinc finger, KRAB, C2H2, transcriptional regulation, DNA-binding, metal binding
LUSC	2	Keratin, peripherin, intermediate
		filament family orphan 1

We next analyzed the lung squamous small cell carcinoma (LUSC) TCGA data set. We performed two–stage medoidshift clustering on the matrix of PCs of CN data, which yielded a result of two clusters. Figure 3b shows the point cloud and cluster assignment, again visualized in the space of the first three PCs. In this case, the clustering performs more poorly. The method captures a subset of tumors that appears to lie along the fringe of the point cloud, but misses an apparent substructure in which a large fraction of the points appear to lie along two distinct two-dimensional surfaces. We suspect the poorer performance in this case is caused by highly uneven sampling of the manifold, with points in the two-dimensonal “wings” heavily concentrated along a single one-dimensional “line” of each wing. We might expect such a geometric structure to occur if each wing represents a mixture of normal cells with a single progression state found in all tumors of that wing and a second, more advanced, progression stage reached by only a minority of tumors.

We again performed Kaplan-Meier analysis on the clusters and observed a test statistic of 3.6413, which is only weakly significant (*p*=0.0339). While the result suggests there is some meaningful separation of the data by survivability, the conclusion must be considered tentative.

The tentative results in the LUSC dataset with respect to survivability lead us to cautiously evalutate the results from ontology analysis. We again used DAVID [51] for amplified regions of the cluster representative for the LUSC dataset, and found terms in the amplified regions’ gene annotations dealing with the keratin family and zinc binding, which have been noted generally as having a role in tumors [56, 57]. Some members of the keratin family have been singled out for study in lung cancers [57], although given the poorer performance of our method on the LUSC dataset, we hesitate to draw conclusions from this correspondence.

## Conclusion and discussion

We have developed and implemented a novel clustering approach for partitioning mixtures of genomic data into submanifolds of interest. Our approach is meant to more accurately estimate of patterns of underlying substructure in tumor genomic data sets indicative of the shared pathways of progression between tumors that can be used to reconstruct heterogeneity within single tumors. The model is designed to exploit the geometric and statistical properties we would expect to arise in genomic point clouds produced by tumors evolving along a common set of possible progression pathways. Tests on synthetic data show the novel two-stage method proposed here is more effective than either pure kernel-based or kernel-free variants of medoidshift clustering at decomposing simplicial complex structure in the presence of ambient noise similar to what we would expect from genomic data. We believe the standard kernel-less method performs poorly because it is not designed for the task of partitioning distinct low-dimensional sub-manifolds of a contiguous higher-dimensional manifold. The negative-weight kernel method solves that problem but at the cost of a very noise-sensitive clustering unsuitable for genomic noise profiles. The proposed two-stage method overcomes these problems by using kernel-less clustering to effectively denoise data followed by the negative-weight kernel to decompose the simplicial complex structure in the denoised data.

The real tumor data shows both the power of the approach and some of its current limitations. The method is effective at decomposing simplicial complex structure evident in the ovarian cancer data, a kind of geometric structure we would predict to arise due to the nature of subtyped progression pathways found in such data. Survival analysis confirms that the derived clusters correspond to a biologically and clinically significant partitioning of tumors. The method performs more poorly on the lung squamous small cell carcinoma data, where a simplicial substructure is visually evident in the data but does not correspond well to the clustering. We attribute this problem to highly uneven sampling of tumors within the sub-manifolds, a problem that is consistent with a model of multistage progression in which tumors may be poorly sampled from late progression stages. The poorer performance in this case suggests avenues for improvement by building expectations from more sophisticated progression models into the clustering objective.

These methods might also be improved by considering other forms of genomic data. The methods are applied here to DNA copy number data and previous methods from our group have used both copy number [21] and gene expression data [19, 33]. Combining distinct data sources on a common tumor, as well as considering alternatives such as genomic methylation data, might be expected to lead to improved mixture separation relative to any one data type. Furthermore, even limited amounts of single-cell genomic data could prove valuable in improving mixture deconvolution of bulk tumor data by more precisely identifying genomic profiles of pure unmixed cell types.

We further note that this clustering is not intended to be a standalone method but rather is meant as part of a broader pipeline for deconvolving mixed genomic data and reconstructing virtual single cell profiles, mixture compositions, and progression pathways [19, 21, 33]. Additional work is still needed to better adapt the present methods for use in the full unmixing process, optimize other steps of that process, and explore ways to better solve the unified inference problem these steps collectively represent. Finally, we note that while our particular interest is analyzing tumor heterogeneity, the methods developed here may have value for analyzing other kinds of genomic data, other forms of heterogeneous cell populations, or deconvolving substructured mixtures in other problem domains.
